# The Concept of Dignity and Its Use in End-of-Life Debates in England and France

**DOI:** 10.1017/S0963180116000050

**Published:** 2016-07

**Authors:** RUTH HORN, ANGELIKI KERASIDOU

**Keywords:** dignity, autonomy, end-of-life decisions, France, England

## Abstract

Dignity is one of the most controversial and yet commonly used terms in debates regarding end-of-life issues. The term “dignity” can take various meanings. For example, it can be used to denote the respect owed to an individual person, or to signify the intrinsic value of humankind as a whole. These two different understandings of dignity inevitably lead to different approaches to end-of-life decisionmaking.

This article explores the meaning of the term “dignity” in two European countries, England and France. Our analysis compares public debates and legislation on end-of-life-related issues in these two countries. We argue that in England dignity is most commonly understood as respect for individual autonomy, whereas in France dignity usually signifies respect for humanity as a whole. We demonstrate that the difference in the conceptualization of the term leads to different ethical, and hence legal and practical, approaches to end-of-life issues and vulnerable patients. Our particular focus is on (1) withdrawing/withholding life-sustaining treatment, (2) respect for patient preferences, and (3) assistance in dying.

Given the difference in the understanding of dignity, and the underlying philosophical approaches, it appears that there is still a long way to go before we can establish common guidelines on end-of-life decisions across Europe and beyond. However, clarifying the use of the term “dignity” in different discussions around Europe could hopefully facilitate this endeavor.

## Introduction

Dignity is a highly debatable concept. Many different meanings have been proposed in ethical and political debates, but a consensus has not been reached.^1^ The lack of a clear definition of dignity has given rise to controversy and confusion. Some authors defend the term,^2,3,4^ whereas others reject it as a useless one in bioethics.^5^ However, dignity remains a prominent concept in international bioethics guidelines and regulations. For example, the 1997 European Convention on Human Rights and Biomedicine,^6^ the UNESCO’s 1997 Universal Declaration on the Genome and Human Rights^7^ and the 2005 Universal Declaration on Bioethics and Human Rights^8^ all invoke human dignity and the obligation to respect it as the basis of restrictions and obligations in biomedical practice.

Respect for dignity is invoked as one of the fundamental principles in moral debates and international guidelines on end-of-life issues. More specifically, Article 5 of the European Convention on Human Rights and Biomedicine presents the obligation to seek patient consent for each health intervention, implying the right to refuse treatment, as a way of respecting dignity and individual freedoms. The convention is endorsed by the majority of European countries, including England and France. These countries have an obligation to implement the directives of the convention nationally. However, national approaches to this implementation can differ markedly. One of the reasons for this discrepancy among countries can be the different meanings of the term “dignity” and the different forms respect for human dignity can take.^9^ It is, therefore, important to explore the different meanings of dignity in an attempt to illuminate its use in the debates^10^ and thus facilitate dialogue among countries.^11^

In this article we focus on two countries, England and France, and the way the international directive regarding respect for dignity has been translated at a national level. We examine the legal approach to end-of-life practices in England and France and also investigate the meaning of the term “dignity” as it appears in public policies and national guidelines.

We argue that in England respect for dignity is mainly understood as respect for autonomy, whereas in France respect for dignity is mainly understood as respect for humanity, solidarity, and public order. We conclude that international guidelines that invoke contentious terms like “dignity” cannot lead to harmonization of policy and practice at a European level and suggest that in order for harmonization of policy and practice to be achieved, the meaning of the term needs to be defined.

## Legal Approaches to End-of-Life Practices in England

Efforts to establish respect for patient dignity have already appeared in international documents. As mentioned previously, the European Convention on Human Rights and Biomedicine emphasizes the obligation to seek free and informed consent from patients, prior to any health intervention (Article 5), as essential to the respect for patient dignity and freedoms (Article 1). Furthermore, the convention requires healthcare providers to show respect for the dignity and freedom of patients by taking into account their wishes regarding end-of-life treatment. However, France and England have adopted different ways of fulfilling this obligation, by ascribing a different legal status to patients’ wishes to refuse treatment.^12^

In English common law, a patient’s right to refuse treatment is based on the principle of bodily integrity, first introduced by William Blackstone in 1765.^13^ As long as patients are duly informed and have understood the consequences of their decision, they do not have to explain in detail why they wish to refuse treatment. Patients can refuse any treatment, even life-sustaining treatment, “for reasons which are rational, irrational, or for no reasons.”^14^ In the case of incompetent patients, advance statements that refuse specific treatments are legally binding in English law, provided the patient had sufficiently comprehended the situation at the time the statement was made.^15^ Only in situations in which there are doubts about the consistency and clarity of a patient’s previously expressed wish may the court decide to overrule the patient’s advance decision to refuse treatment.^16^

Since the Mental Capacity Act of 2005, which came into force in England and Wales in 2007, written advance decisions to refuse treatment have become legally binding also under statute law, provided that certain criteria are met. The 2005 act introduced advance decisions as a way of enhancing the autonomy of patients who had become incompetent. In the absence of such an advance decision, according to section 4 of the act, an incompetent patient’s treatment should be made in his or her “best interests”; this means that the physician should balance medical benefits with the person’s past and present wishes, feelings, beliefs, values, and any other factor the person would consider if he or she were able to do so. The doctor should also take into account the opinion of any other person, such as relatives and close friends, who could contribute to determining what would be in this particular person’s best interests.

## Legal Approaches to End-of-Life Practices in France

In France, the law on patients’ rights [loi sur les droits des patients] of 2002 (law no. 2002-303) introduced the right to refuse treatment.^17^ Many French physicians, however, claimed that it was uncertain as to whether this right included the right to withdraw or withhold life-sustaining treatments.^18^ The 2005 law on patients’ rights and the end of life [loi sur les droits des patients et la fin de vie] (law no. 2005-370) came as an attempt to clarify this confusion. It stipulated that a patient has the right to refuse *any* treatment, including clinically assisted nutrition and hydration (Public Health Code Article L.1111-4).^19^ Although the 2005 law specifies that the doctor has to respect the patient’s wishes, it also states that, where the refusal of treatment endangers the patient’s life, the doctor should “do all that is possible in order to convince the patient” to continue the treatment. It is not specified what doing “all that is possible” actually means, or how far the doctor should go to convince the patient to continue treatment. “In any case,” says the law, “the patient has to repeat his/her decision after a reasonable lapse of time” (Public Health Code Article L.1111-4). As the legal scholar Thouvenin points out, these restrictions express ambivalence toward the recognition of subjective patients’ rights—that is, toward the recognition of the patient as the actual right holder.^20^

The reluctance to rely on the patient’s choice is also apparent in the legal status of advance decisions to refuse treatment in France. The 2005 law stipulates that all competent patients can compose such a document. However, unlike in England, advance decisions are currently not legally binding in France. Article L.1111-11 of the Public Health Code states that they “should be taken into account” by the physician. Prior to taking into account the advance decision, the doctor is advised to consult a colleague, as well as the patient’s representative, family, or close friends. However, it is made clear that it is the physician alone who makes the decision to withdraw or withhold life-sustaining treatment. Despite many attempts in recent years to strengthen patient rights in France, a strong commitment to protect the vulnerable person and to delegate responsibilities to the physician remains the leading element in regulating end-of-life decisions.^21^

Looking at the legal landscape regarding end-of-life practices and advance decisions in England and France, one could argue that although both countries are committed to protecting patient dignity in end-of-life care, their way of achieving this aim is different. In England, patient rights appear to take center stage, whereas in France, the doctors’ duty to safeguard vulnerable individuals is stronger.^22,23^ Because it is the call for respect for dignity that is mainly driving decisions and policies regarding end-of-life treatment, it is important to turn our attention to the way the term “dignity” is commonly used in bioethical reports and medical guidelines in these two countries.

## Dignity in the English End-of-Life Context: Policies and Guidelines

A helpful definition of how human dignity is understood in the English medical context comes from the Nuffield Council on Bioethics. In a 2002 report the council asserts that “an essential ingredient in the conception of human dignity, is the presumption that one is a person whose actions, thoughts and concerns are worthy of intrinsic respect, because they have been chosen, organised and guided in a way which makes sense from a distinctively individual point of view.”^24^ According to this definition, a person’s intrinsic worth, and hence dignity, stems from his or her capacity for autonomy and self-determination. The same understanding of dignity is echoed by the General Medical Council (GMC). The GMC prompts doctors working in end-of-life care to “treat patients as individuals and respect their dignity” by listening and responding to their concerns, by giving patients information in an appropriate way, and by respecting their right to make their own decision.^25^ For both the Nuffield Council and the GMC, treating people with dignity is mainly understood as facilitating, supporting, and promoting their ability and, by extension, their right to choose for themselves and have their choices respected.

In 2008 “distressing reports of people not being treated with dignity and respect [and the fact that] many people do not die where they would choose to”^26^ prompted the publication of a report entitled “End of Life Strategy.” Although other facets of dignity, such as treating the body with dignity or respecting a person’s religious conviction, are mentioned in the “End of Life Strategy” document, the importance of treating someone as an individual with choices and preferences remains the main message of this report.^27^

In addition, the right of the individual to self-determination was successfully defended by Lord Donaldson of Lymington M.R. in the Bland case:

The patient’s interest consists of his right to self-determination . . . , even if it will damage his health or lead to his premature death. Society’s interest is in upholding the concept that all human life is sacred and that it should be preserved if at all possible. It is well established that in the ultimate the right of the individual is paramount.^28^

In the liberal, rights-based context of English culture,^29^ dignity is often associated with self-governance. The rights of patients to take control over their lives and make their own autonomous decisions resonate with England’s philosophical and political tradition. The protection of the individual’s right of liberty against public authorities has been established since the Magna Carta in 1215. As stated elsewhere,^30^ this right has been backed up by important English thinkers such as Locke, who argued that no authority should intervene in the private life of a person,^31^ and Mill, according to whom a person should be free to act autonomously, as long as he or she does not restrain the liberty of others.^32^

As we will see in the next section, France takes a different approach to dignity. In the French context, the person is more embedded in society, and the emphasis is on the equal rights of all members of the community, rather than on individual rights.^33^

## Dignity in the French End-of-Life Context: Policies and Guidelines

The oldest meaning of the word “dignity” refers to a set of qualities and distinctions possessed by people of nobility and those in the higher ranks of society. Kings, ministers, bishops, and doctors had special dignities that came with their roles and positions.^34^ The Declaration of the Rights of Man and of the Citizen (1789), issued as a result of the French Revolution, challenged this definition. It extended dignities to all people regardless of their class or rank, based on the idea that all humans shared a common nature and were equal in the eyes of the law. According to Article 6:

Law is the expression of the general will. . . . It must be the same to all whether it protects or punishes. All citizens, being equal in the eyes of the law, are equally eligible to all dignities and for all public positions and occupations, according to their capacities, and without other distinction than that of their virtues and talents.^35^

As McCrudden shows, respect for all humans beings’ equal dignity is central to French Republicanism, which has been strongly influenced by Jean-Jacques Rousseau’s idea of the social contract.^36^ The republican state—which, according to Rousseau, represents the general will of every citizen—assumes a particular role in protecting equality and guaranteeing the rights of all. Rousseau’s philosophy appears to have contributed to a more egalitarian or communitarian understanding of dignity. Citing Carozza, McCrudden points to the distinctive aspects of the communitarian understanding of dignity, which is known for “‘exhibit[ing] more concern for equality and fraternity, and less exclusive emphasis on liberty’ than that prevalent in North American traditions.”^37^

This particular meaning of dignity and the state’s role in protecting the dignity of its citizens, regardless of their race, age, gender, beliefs or physical condition, is still perceptible in contemporary French legal, ethical and political debates.^38^

In 1994, dignity was introduced in French law as a “principle of constitutional value.” Three so-called laws on bioethics (*lois dites de bioéthique*), governing the protection of personal data, the respect for the human body, and the donation and use of body parts, were adopted in 1994; these laws referred to dignity as the intrinsic value of each person. Since then, Article 16 of the Civil Code has stipulated that the law prohibits any harm to a person’s dignity and guarantees respect for all humans, right from the beginning of their life. Also since 1995, Article 38 of the Code of Medical Deontology (Code de Déontologie Médicale) emphasizes that it is the doctor’s duty to assure a dying patient’s dignity, without, however, intentionally hastening his or her death. This article is integrated into the Public Health Code (Article L.1111-4).

In 2000, the French National Ethics Committee (Conseil Consultatif National d’Ethique) published a report on “end-of-life, ending life, euthanasia” (*fin de vie, arrêt de vie, euthanasie*) in which it stresses the intrinsic value of human dignity, which ought to be protected by the doctor.^39^ According to the committee, physicians are representatives of the society (*corps social*), and their role is to “defend and promote common values, without which there would be neither group nor society.”^40^

Three parliamentary reports on end-of-life issues discuss the different use of the notion of dignity in the debate on end-of-life and euthanasia.^41,42,43^ Dignity can be understood in an individualistic way, denoting each person’s views regarding the value of life. The report acknowledges that many proponents of euthanasia use this definition of dignity to support their arguments. The alternative understanding of dignity, however, signifies the notion of dignity as an intrinsic characteristic of human life, an unalienated quality that all humans share and that cannot be lost or diminished. This is the notion of dignity that opponents of euthanasia usually invoke.

All three reports tend to favor the meaning of dignity as an intrinsic quality of human life. As Vincent Lamanda, the president of the Supreme Court of Appeal maintains: “Human dignity does not imply the liberty to choose one’s own life or death but is the very condition of this liberty . . . the principle of dignity justifies the limitation of a person’s liberty.”^44^

The French National Ethics Committee, in a 2013 report, states that the different meanings of dignity are not a priori contrary to each other.^45^ When a person perceives his or her situation as undignified, the committee, public authority, and society should be mobilized to improve such situations: “The most undignified situation would be to consider the other as being undignified because they are ill, different, alone, unproductive, costly.”^46^ Yet the committee argues that the idea that a person’s dignity might be restored by helping him or her to die infringes on that meaning of dignity that guarantees the equal value of every human being, regardless of his or her condition.

The understanding of dignity as a value that is intrinsic to every human being and that should be protected by public authorities or representatives of society directs not only the debate but also the law and policies on end-of-life practices in France.

Given the differences in the way dignity is understood in England and France, it is worth looking deeper into these two different understandings of the term.

## The Elusive Meaning of Dignity

Dignity is often described as an elusive concept.^47^ In particular, distinguishing between the concepts of autonomy and dignity has presented a significant challenge to many scholars. Some authors have argued that the two notions often collapse into one. Therefore, because autonomy is much easier to define, it has been suggested that the concept of dignity is redundant and should be removed.^48^

Many philosophers have taken it upon themselves to articulate the exact meaning of dignity, and they have proposed a number of different definitions of the term.^49,50,51,52,53,54^ Two particular notions of dignity seem to emerge from looking at England’s and France’s end-of-life debates: dignity as respect for humanity and dignity as respect for autonomy.

### Dignity as Respect for Humanity

Immanuel Kant was the philosopher who put dignity and respect for persons at the center of moral theory. For Kant, human dignity (*Menschenwürde*) is the supreme value that all humans possess in virtue of their humanity—that is, in virtue of their rational nature, being beings capable of rational thinking, autonomous choices, and moral actions.^55^ It is these capabilities, innate to human nature, that render dignity a fundamental and unalienated value of human life. A person’s dignity can be neither lost nor diminished.^56^ As Neumann notes, a person “has all the moral dignity and value a self can have . . . because it operates only according to universal and necessary principles, the same ones for all rational beings.”^57^ It is this ability for self-legislation, rather than the capacity to pursue individual goals, that endows all humans with dignity.

Dignity as respect for humanity is a value that has been used to defend situations in which individual decisions and rights are curtailed. Human dignity was invoked, most famously, in the dwarf-tossing case in France. Although Manuel Wackenheim, the dwarf who was making his living by hiring himself to be tossed, appealed and even took his case to the International Committee on Civil and Political Rights, the committee ruled against him, on the grounds that banning dwarf tossing was necessary for the protection of human dignity.^58^

France’s tendency to surrender individual rights in order to protect societal cohesion and egalitarianism fits with the understanding of dignity that demands respect for humanity as a whole. Respecting dignity means respecting the humanity of every single person who forms the group, rather than the right of each individual to act independently.

### Dignity as Respect for Autonomy

It is the close relationship between the notions of humanity and autonomy that has given rise to the second understanding of dignity we discuss in this article: that of dignity as respect for autonomy.^59^

Autonomy comes from the Greek words *αυτός*, meaning “self,” and *νόμος*, which means “law.” An autonomous person is a self-governing person who decides and is accountable for her actions. Kant described autonomy as the human capacity to govern one’s life in accordance to rational principles.^60^ But, according to Kant, it is practical reason, as exercised through autonomy, that determines our moral obligations toward ourselves and others.^61^ For Mill, however, autonomy is the basis of intrinsic value independent of practical reason. He argued that the capacity for autonomy is one of the main characteristics that differentiate humans from other animals, and that they also endow human life with special moral value.^62^

In Mill’s theory, autonomy is what underlies human dignity:

He who lets the world, or his own portion of it, choose his plan of life for him has no need of any other faculty than the ape-like one of imitation. He must use observation to see, reasoning and judgement to foresee, activity to gather materials for decision, discrimination to decide, and when he has decided, firmness and self-control to hold his deliberate decision. And these qualities he requires and exercises in proportion as the part of his conduct which he determines according to his own judgement and feelings is a large one. It is possible that he might be guided in some good path, and kept out of harm’s way, without any of these things. But what will be his comparative worth as a human being?^63^

The English model of end-of-life care seems to favor the view of dignity as respect for autonomy. The best way to honor human beings and show due esteem for their dignity is to recognize them as autonomous individuals, and allow them to pursue their own goals and dreams. When it comes to patients approaching the end of their lives, the appropriate way of treating them is allowing them to keep pursuing their own individual accounts of the good life until the end, and even beyond.

## Conclusion

Our analysis of the theoretical underpinnings of the English and French attitudes toward end-of-life decisions reveal a difference in the accounts of dignity adopted by the two countries. In the English context, dignity is mainly, yet not exclusively, understood as respect for a person’s autonomy. This has led to laws and practices that safeguard patients’ decisional autonomy, and that recognize the precedence of individual rights over the interests of society.

In the French context, dignity seems primarily to signify respect for humanity. As Renouvier states, the republican ideal “reconciles the interests and the dignity of each individual with the interests and dignity of everyone.”^64^ The emphasis is thus on respecting the intrinsic value of human life, the unalienated property that is equally shared by all human beings. The state’s responsibility is to preserve public order through the protection of human dignity, even if this means limiting individual liberties. Applied to end-of-life decisions and the doctor-patient relationship, patients’ individual choices give way to the doctors’ responsibility to promote social values, such as the protection of patients’ welfare.

Protecting and respecting human dignity is central in many European and international declarations and guidelines regarding end-of-life issues. Given that the role of these guidelines is the alignment of law and practice across countries, understanding the contextual meaning of central concepts such as dignity will help anticipate how these guidelines could be implemented locally.

## Notes

1. For a philosophical analysis of the meaning of dignity, see WoodAW. Kant’s ethical thought. Cambridge: Cambridge University Press; 1999; FosterC. Human dignity in bioethics and biolaw. Oxford: Oxford University Press; 2001; DilleyS, PalpantNJ, eds. Human dignity in bioethics: From worldviews to the public square. New York: Routledge; 2013.

2. SchroederD. Dignity: Two riddles and four concepts. Cambridge Quarterly of Healthcare Ethics 2008;17(2):230–8.1831273810.1017/S0963180108080262

3. HäyryM. Another look at dignity. Cambridge Quarterly of Healthcare Ethics 2004;13(1):7–14.1504590910.1017/s0963180104131034

4. Schroeder D. Dignity: One, two, three, four, five, still counting. *Cambridge Quarterly of Healthcare Ethics* 2010;19(1):118–25.

5. Macklin R. Dignity is a useless concept. *British Medical Journal* 2003(327):1419–20.

6. Council of Europe. Convention for the protection of human rights and dignity of the human being with regard to the application of biology and medicine. Strasbourg: Council of Europe; 1997.

7. UNESCO. Universal declaration on the human genome and human rights; 1997.

8. United Nations. Universal declaration on bioethics and human rights. Geneva: United Nations; 2005.

9. Beširević V. End-of-life care in the 21st century: Advance directive in universal rights discourse. *Bioethics* 2010;24(3):105–12.

10. See note 2, Schroeder 2008.

11. See note 3, Häyry 2004.

12. It is important to note that in England and France the law gives patients the right to refuse but not to request treatment.

13. Blackstone W. *Commentaries on the Laws of England, a Facsimile of the First Edition of 1765–1769*. Chicago: University of Chicago Press; 2002.

14. *Sidaway v. Board of Governors of the Bethlem Royal Hospital* [1985] AC 871.

15. *Re C (Adult: Refusal of Treatment)* [1994].

16. *Re T (Adult: Refusal of Treatment)* [1992] 4 All ER 649.

17. Loi n° 2002-303 du 4 Mars 2002 relative aux droits des malades et à la qualite du systeme de sante. *Journal Officiel*; 2002.

18. Assemblée Nationale. Mission d’information sur l’accompagnement de la fin de vie; 2004.

19. Loi n° 2005-370 du 22 avril 2005 relative aux droits des malades et à la fin de vie. *Journal Officiel*; 2005.

20. Thouvenin D. La loi n° 2005-370 du 22 avril 2005, dite loi Leonetti: la médicalisation de la fin de vie. In: Ferry J.-M., ed. *Fin(s) de vie—Le débat*. Paris: Presses Universitaires de France; 2011:303–68.

21. HornR. Advance directives in English and French law: Different concepts, different values, different societies. Health Care Analysis 2012;22:63–65.10.1007/s10728-012-0210-722555878

22. See note 21, Horn 2012.

23. Of course, like all rules this also has its exceptions. For example, in England, welfare considerations can sometimes outweigh patients’ rights. See *Pretty v. United Kingdom* [2002] 35 EHRR 1; *R (On the Application of Purdy) v. Director of Public Prosecutions* [2009] UKHL 45; *R (On the Application of Nicklinson & Anor) v. A Primary Care Trust* [2013] EWCA Civ 961.

24. Nuffield Council on Bioethics. *Genetics and Human Behaviour: The Ethical Context*. Nuffield Department of Bioethics; 2002, at 121.

25. General Medical Council. *Treatment and Care towards the End of Life: Good Practice in Decision Making*. Department of Health; 2010, at 121.

26. Department of Health. *End of Life Care Strategy: Promoting High Quality Care for Adults at the End of Their Life*. Department of Health; 2008, at 9.

27. *Airedale NHS Trust v. Bland* [1993] AC 789.

28. See note 27, *Airedale NHS Trust v. Bland* 1993.

29. DickensonDL. Cross-cultural issues in European bioethics. Bioethics 1999;13(3–4):249–55.1165723510.1111/1467-8519.00153

30. See note 21, Horn 2012.

31. LockeJ. The Two Treatises of Government. London: Everyman; 1993.

32. MillJS. On Liberty. New Haven, CT: Yale University Press; 2003.

33. Muench R. *Die Kultur der Moderne (2) Ihre Entwicklung in Frankreich und Deutschland*. Frankfurt/Main: Suhrkamp; 1986.

34. Wermiel SJ. Law and human dignity: The judicial soul of Justice Brennan. *William & Mary Bill of Rights Journal* 1998;7(1):223–39.

35. Declaration of the Rights of Man and of the Citizen. In: *Encyclopaedia Britannica*. Online Academic Edition. Encyclopædia Britannica Inc.; 2013.

36. McCrudden C. Human dignity and judicial interpretation of human rights. *European Journal of International Law* 2008;12(4):660.

37. See note 36, McCrudden 2008, at 660.

38. The strong belief in egalitarianism, and the idea that the individual should commit to the good of society, has led to policies that some see as an affront to individual rights of self-expression; see, for example, the dwarf-tossing case (2002) and the law in secularity and conspicuous religious symbols in schools (2004).

39. Comité Consultatif National d’Ethique (CCN). Fin de vie, arret de vie, euthanasie. Ethique et recherche biomedicale [no. 63]. CCN; 2000.

40. See note 39, CCN 2000, at 6.

41. See note 18, Assemblée Nationale 2004.

42. Assemblée Nationale. Mission d’evaluation de la loi n° 2005-370 du 22 avril 2005 relative; 2008.

43. Sicard D. *Rapport de la commission de réflexion sur la fin de vie en France: Penser solidairement la fin de vie*; 2012.

44. See note 42, Assemblée Nationale 2008.

45. Comité Consultatif National d’Ethique (CCN). Fin de vie, autonomie de la personne, volonté de mourir, Ethique et recherche biomedicale [no. 121]. CCN; 2013.

46. See note 45, CCN 2013, at 17.

47. JohnstonJ, EliotC. Chimeras and “human dignity.” The American Journal of Bioethics 2003;3(3):6–8.10.1162/1526516036070671414594463

48. See note 5, Macklin 2003.

49. Spiegelberg H. Human dignity: A challenge to contemporary philosophy. In: Gotesky R, Laszlo E, eds. *Human Dignity: This Century and Next*. New York: Gordon and Breach, Science Publishers; 1970.

50. Mann J. Dignity and health: The UDHR’s revolutionary first article. *Health and Human Rights* 1998;3(2):31–8.

51. PullmanD. Human dignity and the ethics and aesthetics of pain and suffering. Theoretical Medicine and Bioethics 2002;23(1):75–94.1222262210.1023/a:1019521923979

52. Badcott D. The basis and relevance of emotional dignity. *Medicine, Health Care and Philosophy* 2003;6:123–31.

53. Nordenfelt L. The varieties of dignity. *Health Care Analysis* 2004;12(2):69–81.

54. Pullman D. The ethics of autonomy and dignity in long-term care. *Canadian Journal on Aging/La Revue canadienne du vieillissement* 1999;18(1):26–46.

55. Timmons M, ed. *Kant’s Metaphysics of Morals: Interpretative Essays*. Oxford: Clarendon Press; 2002.

56. HillTE. Humanity as an end in itself. Ethics 1980;91(1):84–99.

57. Neumann M. Did Kant respect persons? *Res Publica* 2000;6:285–99, at 294.

58. Kirby EJ. Appeal for “dwarf-tossing” thrown out. *BBC News*; 2002.

59. BeyleveldD, BrownswordR. Human Dignity in Bioethics and Biolaw. Oxford: Oxford University Press; 2001.

60. Kant I. *Grundlegung zur Metaphysik der Sitten*. Stuttgart: Reclam; 1986.

61. See note 57, Neumann 2000.

62. See note 32, Mill 2003.

63. See note 32, Mill 2003, at 125.

64. Renouvier C. *Manuel Républicain de l’homme et du citoyen (1848)*. With introduction by M. Agulhon. Paris: Garnier; 1981, at 93.

